# Association of *FTO* rs1421085 single nucleotide polymorphism with fat and fatty acid intake in Indonesian adults

**DOI:** 10.1186/s13104-021-05823-1

**Published:** 2021-11-07

**Authors:** Athraa Alaulddin Al-Jawadi, Lidwina Priliani, Sukma Oktavianthi, Clarissa A. Febinia, Mulianah Daya, I Made Artika, Safarina G. Malik

**Affiliations:** 1 Department of Biochemistry, Faculty of Mathematics and Natural Sciences, Bogor Agricultural University, Kampus IPB Dramaga, Jl. Raya Dramaga, Bogor, 16680 West Java Indonesia; 2 Eijkman Institute for Molecular Biology, Ministry of Research and Technology/National Research and Innovation Agency, Jl. Diponegoro No. 69, Jakarta, 10430 Indonesia; 3 Department of Nutrition, Faculty of Medicine, Universitas Indonesia/Cipto Mangunkusumo National General Hospital, Jakarta, Indonesia

**Keywords:** *FTO*, rs1421085, Fatty acid, Fat intake, Obesity, Indonesia

## Abstract

**Objective:**

Recent studies showed that genetic polymorphisms in the fat mass and obesity-associated gene (*FTO*) were associated with obesity and dietary intake. In this study of 71 adults in Jakarta, Indonesia, we investigated *FTO* rs1421085 association with body mass index (BMI), macronutrient intake, and fatty acid intake. The association was evaluated using linear regression analyses assuming co-dominant, dominant, recessive, over-dominant, and additive genetic models.

**Results:**

Only individuals with the CC genotype had a considerably higher BMI (*p* < 0.001), which indicates a recessive genetic trait, but the incidence for this genotype is low (68 TT + TC vs. 3 CC). Individuals with the minor C allele had an estimated increase of fat intake by 3.45–4.06% across various genetic models (dominant: *p* < 0.010, over-dominant: *p* < 0.030, additive: *p* < 0.010). Subjects with TC/CC genotypes had increased dietary monounsaturated fatty acid (MUFA; 1.14%, *p* = 0.046) and saturated fatty acid (SAFA; 2.06%, *p* = 0.023) intakes, compared to those with the TT genotype. In conclusion, our study provided evidence for the association between *FTO* rs1421085 risk allele with higher BMI and individual preferences for consuming more fat, MUFA, and SAFA. This study highlights the important role of *FTO* gene in food preference, and its influence on body weight.

**Supplementary Information:**

The online version contains supplementary material available at 10.1186/s13104-021-05823-1.

## Introduction

WHO reported that 13% of the global population of adults were obese in 2016 [1], and it is projected that 1 in 5 adults will be obese in 2025 [2]. Obesity manifestation is a complex interaction between overnutrition, sedentariness, and genetic factors [3]. Obesity in Indonesia has increased significantly between 2007 (10.5%) and 2018 (21.8%) [4, 5]. There is a growing interest in the genetic predisposition of obesity in Indonesia since mortality due to obesity comorbidities have risen to the nation’s top [6–8].

The association between the fat mass and obesity-associated (*FTO*) and obesity is well-documented, particularly in Caucasian populations, albeit the overall risk is modest [9–15]. Single nucleotide polymorphism (SNPs) in the *FTO* gene were associated with other obesity traits (e.g., body weight, leptin levels, body fat, waist circumference) [9–13, 15–17]. East and South Asian populations showed comparable associations for common *FTO* variants (notably rs9939609, rs1558902, and rs1421085) [16–21], but the effect size may vary depending on ethnicity and dietary intake [22, 23]. Concurrent associations between *FTO* variants, obesity, and dietary intake have been noted in several ethnic groups [22–27], including our previous report that *FTO* rs9939609 TT genotype was associated with obesity and preference for a high-fat diet in adult individuals from Jakarta [28].

Expression of the *FTO* gene also affects various parts of the brain that regulate energy balance and appetite [3, 29, 30]. *FTO* mutation rs9939609 is associated with not only obesity, but also macronutrient intake, that includes: carbohydrates [26, 31], protein [22], polyunsaturated fatty acid (PUFA), and saturated fatty acid (SAFA) [32]. We expect that a similar influence for *FTO* rs1421085 which has a high linkage disequilibrium with *FTO* rs9939609 [28]. Several studies reported that *FTO* rs1421085 is associated with both obese-related phenotypes and dietary macronutrient intake [16, 17, 33]. In vivo and in vitro model studies showed that *FTO* rs1421085 can alter the binding of transcriptional repressors in nearby regions, affecting the expression of genes linked to adipocyte thermogenesis and food intake [34, 35].

Understanding the interaction between diet and *FTO* variants in Indonesia may provide valuable insights for obesity management since Indonesian cuisine is naturally fatty, owing to the generous use of coconut milk and palm oil [36–38]. Dietary fat and SAFA intake in Indonesia was the highest among countries across the globe (31.9% and 20.9%, respectively) [39].

As a continuation of our previous study [28], we performed genotyping for *FTO* rs1421085 and analyzed its association with obesity and dietary intake. We hypothesize that individuals with the minor risk allele have higher Body Mass Index (BMI), higher fat intake, and distinct fatty acid intake profile (PUFA, monounsaturated fatty acid/MUFA, and SAFA). Our findings provided valuable insights into the influence of the *FTO* rs1421085 risk allele on BMI and individual preferences for consuming more fat, particularly MUFA and SAFA.

## Main text

### Methods

#### Study design and subjects

We performed a follow-up case–control genetic association study of *FTO* rs1421085 on a study population from Daerah Khusus Ibukota (DKI) Jakarta, Indonesia. Study enrollment and collection of informed consent were done as described previously by Daya et al*.* [28]. We obtained ethical approval from the Medical Research Ethics Committee of the Faculty of Medicine, Universitas Indonesia (No. 1137/UN2/F1/ETIK/2017, protocol number 17-12-1212). We collected age, gender, and residence data at enrollment. We calculated BMI as body weight (kg) per squared height (m^2^). We used the International Obesity Task Force (IOTF) definitions for Asian obesity (BMI ≥ 25 kg/m^2^) and non-obesity (BMI < 23 kg/m^2^) [40]. We obtained dietary data with questionnaires and calculated dietary intake using NutriSurvey 2007 as previously described [28, 41].

#### Genotyping

We assessed the quantity and purity of archived DNA samples from our previous study [23] using a Nanodrop-1000 (Perkin Elmer Biosystem, USA). *FTO* rs1421085 was genotyped using amplification-refractory mutation system polymerase chain reaction (ARMS PCR) as described by Priliani et al*.* [42] in a GeneAmp PCR System 9700 (Applied Biosystems, USA). PCR products were separated in 2% agarose gel electrophoresis (Lonza, Basel, Switzerland) and visualized using the Gel Doc XR Imaging System (BioRad, USA). The primer sequences are in Additional file 1: Table S1.

#### Statistical analysis

We used R version 4.0.3 for all statistical tests (packages: “stats”, “genetics”, “Rfit”). Data distribution normality was assessed using the Shapiro–Wilk test. Differences in anthropometric values and dietary intake between obese and non-obese participants were calculated using the unpaired t-test for normally distributed variables and the Mann–Whitney U test for non-normal variables. Allele and genotype frequencies were calculated and Hardy–Weinberg equilibrium test was performed. Association analyses were carried out using either a linear regression analysis (normal variables) or a rank-based linear regression analysis (non-normal variables), adjusted for age and sex (formula: outcome ∼ SNP + age + sex). We assessed the following genetic models: co-dominant (factorial variables: TT = 0, TC = 1, and CC = 2), dominant (TT = 0, TC + TT = 1), recessive (TT + TC = 0, CC = 1), over-dominant (TT + CC = 0, TC = 1), and additive (continuous variables: TT = 0, TC = 1, and CC = 2). Upper and lower intervals were calculated at 95% confidence levels with no adjustments for multiple comparisons. The value of *p* < 0.05 was considered statistically significant.

### Results

#### Baseline characteristics

Available archived DNA samples consisted of 36 non-obese and 35 obese individuals aged 19–52 years. The difference in gender proportions between the non-obese and obese group was significant (51 female, 29 male, χ^2^ = 7.36, *p* = 0.013). The BMI (median) for the obese and non-obese groups were 31.86 and 20.86, respectively (*p* < 0.001). Age and dietary parameters are comparable in obese and non-obese samples (all *p* > 0.05, Additional file 1: Table S2).

#### Genotype and allele distributions

The *FTO* rs1421085 genotype and allele distribution are in Table 1. The minor allele frequency (MAF) was 22% in total samples (n = 71), 24% in obese samples (n = 35), and 19% in non-obese samples (n = 36). The genotype distribution did not depart from Hardy–Weinberg equilibrium for total, obese, and non-obese samples (all *p* = 1). The frequency of CC genotype was notably low.

**Table 1:** FTO rs1421085 genotype and minor allele frequency (MAF), Hardy–Weinberg equilibrium test

Genotype	Total, n (%)	Obese, n (%)	Non-obese, n (%)
	n = 71	n = 35	n = 36
TT	43 (60.6%)	20 (57.1%)	23 (63.9%)
TC	25 (35.2%)	13 (37.1%)	12 (33.3%)
CC	3 (4.2%)	2 (5.7%)	1 (2.8%)
MAF	22%	24%	19%
p-HWE	1	1	1

*MAF* minor allele frequency, *HWE* Hardy–Weinberg equilibrium

#### Associations of *FTO* rs1421085 with BMI and diet

By evaluating various genetic models, we found several notable associations between *FTO* rs1421085 with BMI, macronutrient intake, and fatty acid intake (Table 2). BMI is greater by 12.58 kg/m^2^ (*p* = 0.001) and 12.38 kg/m^2^ (*p* < 0.001) in individuals with the CC genotype under the co-dominant and recessive models, respectively. These individuals also reported lower carbohydrate intake by 7.84% (*p* = 0.029) and 7.59% (*p* = 0.031) under the co-dominant and recessive models, respectively. This data suggests that *FTO* rs1421085 associations with higher BMI and lower carbohydrate intake might be a recessive trait that showed only in individuals with CC genotype. However, we consider these findings inconclusive since the CC genotype frequency was very low (n = 3).

**Table 2:** Associations between FTO rs1421085, macronutrient intake, and dietary fatty acids intake in various genetic models

Genetic model	*n*	BMI (kg/m^2^)		Carb. intake (%)		Protein intake (%)		Fat intake (%)		PUFA intake (%)		MUFA intake (%)		SAFA intake (%)	
		Coef. (95% CI)	*p*	Coef. (95% CI)	*p*	Coef. (95% CI)	*p*	Coef. (95% CI)	*p*	Coef. (95% CI)	*p*	Coef. (95% CI)	*p*	Coef. (95% CI)	*p*
**Co-dominant**															
TT	43	Ref.		Ref.		Ref.		Ref.		Ref.		Ref.		Ref.	
TC	25	0.47 (− 2.66–3.6)	0.766	− 0.70 (− 3.64–2.25)	0.639	0.27 (− 1.18–1.73)	0.709	3.87 (0.69–7.05)	**0.018**	0.45 (− 0.72–1.63)	0.444	1.13 (− 0.03–2.29)	0.055	1.77 (− 0.06–3.6)	0.058
CC	3	12.58 (5.15–20.01)	**0.001**	− 7.84 (− 14.84 to − 0.83)	**0.029**	0.77 (− 2.68–4.22)	0.657	5.63 (− 1.92–13.18)	0.141	0.84 (− 1.96–3.64)	0.551	1.27 (− 1.48–4.03)	0.360	4.49 (0.14–8.84)	**0.043**
**Dominant**															
TT	43	Ref.		Ref.		Ref.		Ref.		Ref.		Ref.		Ref.	
TC + CC	28	0.70 (− 2.27–3.67)	0.640	− 1.47 (− 4.38–1.44)	0.316	0.35 (− 0.98–1.68)	0.602	4.06 (1.01–7.11)	**0.010**	0.54 (− 0.59–1.67)	0.341	1.14 (0.02–2.26)	**0.046**	2.06 (0.29–3.83)	**0.023**
**Recessive**															
TT + TC	68	Ref.		Ref.		Ref.		Ref.		Ref.		Ref.		Ref.	
CC	3	12.38 (5.3–19.46)	**< 0.001**	− 7.59 (− 14.47–-0.71)	**0.031**	0.70 (− 2.7–4.1)	0.681	4.24 (− 3.48–11.97)	0.277	0.71 (− 1.97–3.39)	0.599	0.70 (− 2.31–3.71)	0.644	3.86 (− 0.53–8.24)	0.084
**Over-dominant**															
TT + CC	46	Ref.		Ref.		Ref.		Ref.		Ref.		Ref.		Ref.	
TC	25	0.20 (− 2.84–3.25)	0.894	− 0.20 (− 3.2–2.8)	0.895	0.22 (− 1.24–1.68)	0.766	3.51 (0.34–6.68)	**0.030**	0.41 (− 0.73–1.54)	0.477	1.06 (− 0.14–2.26)	0.083	1.48 (− 0.37–3.33)	0.115
**Additive**	71	1.17 (− 1.38–3.72)	0.363	− 1.99 (− 4.42–0.45)	0.109	0.32 (− 0.84–1.48)	0.586	3.45 (0.86–6.04)	**0.010**	0.43 (− 0.51–1.38)	0.365	0.86 (− 0.1–1.82)	0.078	1.96 (0.47–3.45)	**0.011**

BMI: Body Mass Index (kg/m^2^); Carb.: carbohydrate; PUFA: polyunsaturated fatty acid; MUFA: monounsaturated fatty acid; SAFA: saturated fatty acid; Coef.: regression coefficients; Ref.: indicate the reference genotype in a particular genetic model; *p*: *p*-values obtained through linear model analyses for normally distributed variables (carbohydrate, fat, and SAFA intake) or rank-based linear model analyses for non-normal variables (BMI, protein, PUFA, and MUFA); CI: confidence interval

All analyses were adjusted for age and sex. Significant associations were marked in bold (*p* < 0.05)

No associations with *FTO* rs1421085 were found for protein intake, but associations with fat intake (%) were significant (Table 2). Individuals with TC genotype showed a slightly higher fat intake by 3.87% (*p* = 0.018) and 3.51% (*p* = 0.030) under the co-dominant and over-dominant models, respectively. The increase was comparable under the dominant (TC + CC: 4.06%, *p* = 0.010) and additive model (3.45%, *p* = 0.010). Given these findings, *FTO* rs1421085 association with higher fat intake appeared as a dominant or an additive trait since it was overall stronger in individuals with the minor risk allele.

There were notable associations with MUFA and SAFA intake (Table 2). The association with MUFA was minor; individuals with TC + CC genotypes intake were projected with 1.14% (*p* = 0.046) higher MUFA intake only in the dominant model, whereas the association under the co-dominant, over-dominant, and additive models were marginal (*p* < 0.100). The co-dominant model showed that individuals with TC and CC genotype had respectively greater SAFA intake by 1.77% (*p* = 0.058) and 4.49% (*p* = 0.043) when compared to individuals with TT genotype. The dominant (TC + CC: 2.06%, *p* = 0.023) and additive model (1.96%, *p* = 0.011) showed comparable results. This data showed that the association with SAFA is likely a dominant or an additive trait, and it is in line with our analysis of fat macronutrient intake.

### Discussion

The rapid increase of obesity incidence and mortality caused by its comorbid conditions have gathered concerns over genetic predispositions to obesity in Indonesia. Recent studies have reported interactions between *FTO* SNPs with dietary factors [22–32]. There is a scientific need to investigate the link between the *FTO* gene and dietary intake and further elucidate the underlying mechanisms of their interaction. *FTO* genetic variants with obesity can differ by ethnicity and dietary preference [16, 22, 23, 25, 28]. In this study, we performed a genetic association study employing various genetic models to assess the impact of *FTO* rs1421085 on obesity and dietary preference in individuals residing in Jakarta, Indonesia. We found that *FTO* rs1421085 is a common mutation in our studied population and the minor risk allele is associated with BMI, higher intake of dietary fat, MUFA, and SAFA.

The minor allele frequency of *FTO* rs1421085 in our studied population (22%) is lower than our finding in Balinese (41%) [42]. The frequency is only slightly higher than Asian populations (13–14%, excluding South Asians), but lower than South Asian (31%) and European populations (42%) [43, 44]. Our sample size was admittedly small compared to these studies, but the disparity might also be unique to this specific population due to Indonesia’s population diversity [45], natural selection, genetic drift, and mutation [46]. A further study is required to confirm the allelic distribution in Jakarta.

Our assessment of various genetic models indicates that *FTO* rs1421085 association with BMI is a recessive trait, but the frequency of the heterozygous CC genotype is small. A study employing 84 individuals from Tehran has a similar sample size to ours, and it did not find any association between *FTO* rs1421085 and BMI [23]. Given that Asian populations have a low frequency of the risk allele, it is likely that a dataset smaller than a few hundred samples might not be sufficient to assess the association.

As expected, we found that *FTO* rs1421085 was associated with higher dietary fat intake, particularly with SAFA intake. There was also a minor association with MUFA intake. This data supports the findings in an adult Caucasian population, in which the risk allele of *FTO* rs1421085 was found associated with perceived hunger, higher intake of high-fat foods, and increased body weight [47]. Our findings support our previous assessment of the *FTO* rs9939609 risk allele in the same studied population that showed an association to higher fat intake [23]. *FTO* rs9939609 and rs1421085 have high linkage disequilibrium in our previous study of a Balinese population [29].

Our findings support that *FTO* gene expression is associated with fatty acid intake (MUFA and PUFA) [48]. *FTO* rs1421085 can influence the expression of several genes linked to food intake and appetite [34, 35], which might explain why we found associations between the risk allele, dietary fat, and SAFA intake under the additive genetic model that imply incremental allelic influence. Notably, *FTO* can impact signaling pathways that regulate eating behavior, satiety, stress response, mood, and perception of food [29, 30, 49, 50]. Our findings added to this body of knowledge by confirming the association between *FTO* rs1421085 with dietary fat intake in a small population in Jakarta.

The direction of causality of these associations is not confirmed. However, these findings are compelling evidence that food preference (e.g. dietary fatty acids) can be influenced by the *FTO* gene and may subsequently affect body weight. Understanding this interaction would be valuable in developing a personalized nutrition strategy to combat obesity.

### Conclusions

*FTO* rs1421085 TC + CC genotypes were positively associated with BMI, higher dietary fat, MUFA, and SAFA in individuals from Jakarta. Future studies should investigate the mechanisms of actions that underlie the correlation between *FTO* and dietary preference and its impact on individual weight management.

## Limitations

The sample size for this study was limited; so, a follow-up study with a larger sample size is required. There might be geographical and gender bias since we only sampled 71 individuals in Jakarta, of which the majority were female. A more comprehensive anthropometric and clinical data is recommended for future studies to assess other obesity-related parameters (e.g., waist circumference, leptin levels).

## Supplementary Information

**Additional file 1: Table S1.** Primers used for ARMS-PCR detection. Oligonucleotide sequences were obtained from Priliani et al*.* [42]. Fin: forward inner primer; Rin: reverse inner primer; Fout: forward outer primer; Rout: reverse outer primer. **Table S2.** Baseline characteristics of obese and non-obese subjects. Demographic, anthropometric, and dietary data are presented as mean ± SD for normally distributed variables and median (Quantile 1–Quantile 3) for abnormally distributed data. Differences between groups assessed using t-test for normally distributed variables, or Mann–Whitney U test for non-normal variables. Significant differences (p < 0.05) are marked in bold.

## Data Availability

The dataset analyzed in this study is available upon reasonable request from the corresponding author.

## Abbreviations

ARMS-PCR: Amplification-refractory mutation system-polymerase chain reaction
BMI: Body mass index
FTO: Fat mass and obesity-associated
HWE: Hardy–Weinberg equilibrium
LD: Linkage disequilibrium
MAF: Minor allele frequency
MUFA: Monounsaturated fatty acid
PUFA: Polyunsaturated fatty acid
SAFA: Saturated fatty acid
SNPs: Single nucleotide polymorphisms
