# Supplementary material for: Association of FTO rs1421085 single nucleotide polymorphism with fat and fatty acid intake in Indonesian adults
Source: BMC Res Notes. 2021 Nov 7;14:411. doi: 10.1186/s13104-021-05823-1 (PMC8574008; doi:10.1186/s13104-021-05823-1)
Supplement: Supplementary file 1 — Additional file 1: Table S1. Primers used for ARMS-PCR detection. Oligonucleotide sequences were obtained from Priliani et al. [42]. Fin: forward inner primer; Rin: reverse inner primer; Fout: forward outer primer; Rout: reverse outer primer. Table S2. Baseline characteristics of obese and non-obese subjects. Demographic, anthropometric, and dietary data are presented as mean ± SD for normally distributed variables and median (Quantile 1–Quantile 3) for abnormally distributed data. Differences between groups assessed using t-test for normally distributed variables, or Mann–Whitney U test for non-normal variables. Significant differences (p < 0.05) are marked in bold. [file 13104_2021_5823_MOESM1_ESM.docx]

Table S1 Primers used for ARMS-PCR detection

| SNP | Primers | Fragments |
| --- | --- | --- |
| rs1421085 | Fin: 5’- TAGCAGTTCAGGTCCTAAGGCATTAT-3’  Rin: 5’- ACAAATTCTCATCAGACACTTAATCACTG-3’  Fout: 5’- TTTAGGTTGTAATGAAGTTTTAGGCCTC-3’  Rout: 5’- ATCAGGTTAAATAAATGCTTCTGGACAG-3’ | TT: 446 bp, 205 bp  TC: 446 bp, 295 bp, 205 bp  CC: 446 bp, 295 bp |

Oligonucleotide sequences were obtained from Priliani et al (2020). Fin: forward inner primer, Rin: reverse inner primer, Fout: forward outer primer, Rout: reverse outer primer

Table S2 Baseline characteristics of obese and non-obese subjects

| **Variable** | **Obese (n=35)** | **Non-obese (n=36)** | ***p*** |
| --- | --- | --- | --- |
| **Age** | 33 (27.5 – 39) | 31 (27.5 – 34.6) | 0.430 |
| **Gender n (%)** |  |  |  |
| Male | 15 (42.9 %) | 5 (13.9 %) |  |
| Female | 20 (57.1 %) | 31 (86.1 %) |  |
| **BMI (kg/m²)** | 31.86 (28.10 – 35.39) | 20.86 (19.48 – 21.39) | **<0.001** |
| **Energy intake (kcal)** | 1595 (1259 – 1980) | 1588 (1258 –1937) | 0.666 |
| **Macronutrients** |  |  |  |
| Total carbohydrate (%) | 43.17 ± 6.11 | 40.93 ± 5.62 | 0.112 |
| Total protein (%) | 14.75 (12.88 – 16.12) | 14.75 (13.44 – 17.06) | 0.267 |
| Fat intake (%) | 34.53 ± 6.10 | 33.33 ± 6.83 | 0.437 |
| **Fatty acids** |  |  |  |
| PUFA (%) | 5.19 (4.02 – 7.26) | 6.65 (5.08 – 7.50) | 0.141 |
| MUFA (%) | 8.20 (7.31 – 9.84) | 8.38 (7.09 – 10.40) | 0.870 |
| SAFA (%) | 17.12 ± 3.38 | 15.55 ± 3.98 | 0.076 |

Data are presented as mean ± SD for normally distributed data and median (Quantile 1 – Quantile 3) for non-normal variables. Differences between groups assessed using t-test for normally distributed variables, or Mann-Whitney U test for non-normal variables. Significant differences (*p*<0.05) are marked in bold.
